# Phosphodiesterase Type 5 Inhibitors and sudden sensorineural hearing loss

**DOI:** 10.5935/1808-8694.20130133

**Published:** 2015-10-08

**Authors:** Monique Antunes de Souza Chelminski Barreto, Fayez Bahmad

**Affiliations:** a MSc in Health Sciences from the FCS - UNB; Audiologist, Specialist by the UFPE Specialist in Psycho-Pedagogy from the UFRJ; Audiologist of the Hospital Foundation of the Federal District- GDF; Ph.D. Student in Health Sciences, School of Health Sciences, UNB; b PhD in Medical Sciences from the School of Medicine of UNB; Professor at the Graduate Program, School of Health Sciences, UNB

**Keywords:** phosphodiesterase inhibitors, sudden hearing loss, tinnitus

## Abstract

Phosphodiesterase type 5 Inhibitors, such as sildenafil, vardenafil and tadalafil have been increasingly used today and some of the users have developed sudden sensorineural hearing loss.

**Objective:**

To present two patients with sudden deafness developed after an occasional use of the drug and review studies on the use of phosphodiesterase type 5 inhibitors and sudden hearing loss.

**Method:**

Analytical study of two cases and review of the subject matter in the Pubmed/Medline and Bireme databases using the keywords: phosphodiesterase type 5 inhibitors and sudden deafness and its correlates in the English language.

**Results:**

The patients analyzed are young without additional disorders, using phosphodiesterase type 5 inhibitors, and after combination treatment for sudden hearing loss only one had hearing improvement. We found nine scientific studies and reviewed preclinical studies, clinical trials, prospective and cross-sectional investigations.

**Conclusion:**

Increased occurrence in clinical practice and scientific reports in the literature suggest that the phosphodiesterase type 5 inhibitors are considered a risk factor for sudden deafness. Further studies with larger samples and control groups are needed for better assessing this association.

## INTRODUCTION

Ototoxic drugs may cause balance and hearing disorders, modifying cochlear and vestibular functions. The most common known ototoxic drugs are aminoglycoside antibiotics; antineoplastic agents, particularly platinum-based (cisplatin and carboplatin); as well as some loop diuretics: furosemide, salicylic acid and quinine^1^.

In the first case report associating the use of phosphodiesterase type 5 inhibitor (PDE5i) with hearing loss in 2007, the authors attributed this disorder to a probable ototoxic effect of the drug used (sildenafil) and pointed out that there is an extensive list of ototoxic drugs such as aminoglycosides, antibiotics and other medications, and that was the first case correlating the use of sildenafil and sudden-onset hearing loss^2^.

Phosphodiesterase type 5 inhibitors (PDE5i) such as sildenafil, vardenafil and tadalafil are drugs used to treat erectile dysfunction, but sildenafil is also indicated for pulmonary hypertension. Common adverse effects include headache, facial flushing, nasal congestion, dyspepsia and nausea. Recently, PDE5i have been associated with adverse effects involving vision, and there is emerging evidence suggesting that such drugs can be also responsible for hearing disorders, increasing the risk of sudden hearing loss^3^.

Recent concerns about this class of drugs and the emergence of cases of sudden sensorineural hearing loss (SSNHL) resulted in a requirement of the FDA (Food and Drug Administration) - U.S agency that regulates food and pharmaceutical products - for a more rigorous labeling. Based on these reports, researchers stressed the need to investigate the potential relationship between sudden sensorineural hearing loss and use of this class of drugs^4^.

Such drugs are commercially known as sildenafil citrate (Viagra® and Revatio®, Pfizer), vardenafil (Levitra®, distributed by Bayer Pharmaceuticals, Schering-Plough and GlaxoSmithKline), and tadalafil (Cialis®, Eli Lilly). Sildenafil was the first PDE5i licensed for erectile dysfunction treatment and has been widely prescribed since it was FDA-approved in the United States^5^. Though used intermittently, sometimes sildenafil must be taken continuously and in large doses. The recommended dose of sildenafil citrate for the treatment of erectile dysfunction is 25–100 mg per day^6^.

Sudden deafness is characterized as a sensorineural hearing loss of sudden onset and unknown cause. There may be inner ear and/or central auditory pathways involvement, of variable intensity and frequency, ranging from a mild feeling of stuffed ear, total hearing loss, and tinnitus in approximately 80% of the cases7., 8., 9.. Tinnitus may vary in intensity and occurs in 70% of the cases, and it may recede or persist longer than the hearing loss^10^.

The hearing loss sensation can range from mild to very severe, and it may yield to spontaneous recovery in 25% or more cases. Sudden hearing loss progression behavior is quite variable, with total or partial spontaneous recovery; and it may leave irreversible auditory sequelae^7^.

In a histopathological study of the temporal bone, after an episode of sudden hearing loss, there was great loss of cochlear neurons, and the organ of Corti and outer hair cells were missing in most of the cochlear basal turn, justifying, in that case, the hearing loss in the high frequencies^11^.

Recent evidence has suggested that phosphodiesterase type 5 inhibitors may be responsible for sensorineural hearing loss of sudden onset2., 3., 6..

Given the relevance of the proposed topic and the fact that there are no publications in Portuguese, we became interested in carrying out this study, in order to present two cases of patients with sudden sensorineural hearing loss who were using phosphodiesterase type 5 inhibitors, and revisit studies on this correlation.

## METHOD

We carried out an analytical study of two cases of young patients without other disorders, who were using phosphodiesterase type 5 inhibitors and developed sudden sensorineural hearing loss without hearing improvement after combination therapy (oral steroids - OS and intratympanic steroid injection ITS), for hearing and tinnitus. Patients signed an informed consent form (ICF) and the study was approved by the Research Ethics Committee of the University of Brasília (UNB), under protocol number 132/12.

The search strategy employed in the literature review was guided using the keywords indexed in DecS (Descriptors in Health Sciences) in Portuguese and in MeSH (Medical Subject Headings) in English: sudden hearing loss and phosphodiesterase inhibitors and their correlates in English: sudden deafness and phosphodiesterase inhibitors. For this, we used the PubMed and Bireme/MedLine databases, and analyzed all papers published in indexed journals, including preclinical, clinical, prospective and cross-sectional studies.

In this study, we considered only those papers published from 1998 to June 2013, a total of 15 years, and the last manual search conducted in electronic databases occurred in June 2013.

## RESULTS

For the two cases presented, Patient 1, aged 37, sought medical attention as soon as his mild vertigo and hearing loss symptoms started, and he was immediately subjected to treatment with the use of combination therapy for sudden hearing loss, that is, oral corticosteroids (OS - prednisolone, 1 mg/kg/day for 10 days with a reduction of 10 mg every two days) associated with intratympanic steroid injection three times every other day (ITS - Methylprednisolone 0.7 ml of a 40 mg/ml solution, Solumedrol®), and the treatment was uneventful.

In the first pure tone audiometry the patient did not have hearing thresholds in the left ear. He was also submitted to laboratory tests and MRI. He was submitted to distortion products evoked otoacoustic emissions and auditory evoked potential tests, and had results consistent with profound sensorineural hearing loss, or no response. There was no improvement even after combined therapy (OS + ITS) in audiometric tests performed one month after treatment.

Patient 2, aged 43, presented hearing loss, tinnitus and severe vertigo as initial symptoms. He was submitted to laboratory tests and MRI, as well as hearing assessment by pure tone audiometry, otoacoustic emissions and Evoked Auditory Brainstem Response, with improvements in his hearing thresholds.

On tinnitus, we used the Visual Analogue Scale (VAS) for discomfort and intensity as a subjective assessment and there was an improvement in score (Table 1); Patient 1, being classified as severe to moderate after combined therapy and Patient 2 being moderate to mild after therapy. In the Tinnitus Handicap Inventory (THI), we noticed that Patient 1 had a change in category, from catastrophic to severe; and Patient 2 obtained improvement in score, moving from severe to mild (Table 2).

**Table 1:** Description of the two patients using phosphodiesterase type 5 inhibitors who developed sudden hearing loss.

	Age (years)	Gender	Treatmentemployed	Hearing thresholds before the Treatment (Mean Tritonal)	Hearing thresholds after the Treatment (Mean Tritonal)	Vertigo associated with SNHLSO before combined treatment	Vertigo associated with SNHLSO aftercombinedtreatment	Tinnitus before combined treatment (EVA)	Tinnitus after combined treatment (EVA)
									
				> 110 dB SPL	>110 dBSPL				
Patient 1	37	M	OS + ITS	Profound	Profound	Yes	No	10 (Intense)	07 (Moderate)
				sensorineural	sensorineural				
				hearing loss	hearing loss				
				85 dBSPL	27 dB SPL				
Patient 2	43	M	OS + ITS	Severesensorineural	Mildsensorineural	Yes	No	07 (Moderate)	02 (Mild)
				hearing loss	hearing loss				

SPL: Sound pressure level; VAS: Visual Analogue Scale - Intensity of Tinnitus - 0–2: Mild; 3–7: Moderate; 8–10: Severe.

**Table 2:** Description of the results obtained in the Tinnitus Handicap Inventory (THI) upon monitoring the patients.

	Age(years)	Beforecombinedtreatment	1st week aftercombinedtreatment	1st month aftercombinedtreatment
Patient 1	37	96 (level 5) catastrophic	64 (level 4) severe	60 (level 4) severe
Patient 2	43	58 (level 4) severe	36 (level 2) severe	30 (level 4) mild

Level 1 (score 0–15 - discreet); Level 2 (score 18–36 - mild); Level 3 (score 38–56 - moderate); Level 4 (score 58–76 - severe); Grade 5 (score 78–100 - catastrophic).

We found nine papers. The first publication correlating the use of phosphodiesterase type 5 and sudden hearing loss is from 2007 (Chart 1).

**Chart 1:** Studies correlating the use of phosphodiesterase type 5 and sudden sensorineural hearing loss.

Study site and year	Author	Study type	Population		Assessment method	
				PTA	TEOAE/DPEOAE	ABR
2007India	Mukherjee & Shivakumar^2^	Case study	44-year-old man	X	X	X
2008South Korea	Hong et al.^6^	Experimental study	Albino guinea pigs		X	X
2009USA	Maddox et al.^4^	Case study and review	25 men	NA	NA	NA
2009Turkey	Okuyucu et al.^12^	Prospective observational study	18 men	X		
2010USA	McGwin^13^	Cross-sectional cohort (Epidemiological)	11,525 men	NA	NA	NA
2010USA	Snodgrass etal.^3^	Case study	57-year old man	X		
2011United Kingdom	Khan et al.^14^	Review	47 men	NA	NA	NA
2012Turkey	Bakir et al.^15^	Experimental study	Albino guinea pigs		X	
2013India	Thakur et al.^16^	Prospective observational study	25 men	X		X

PTA: Pure tone audiometry; DPEOAE/TEOAE: Distortion Product Evoked Otoacoustic Emissions/Transient evoked otoacoustic emissions;

ABR: Auditory Brainstem Response, NA: Not applicable.

## DISCUSSION

The effects of phosphodiesterase type 5 inhibitors in the cochlea are still uncertain due to the need for more studies with larger groups and a control group, and there is a need for otolaryngologists to add to their anamneses the possibility that patients with this type of hearing loss may be using this class of drugs, since there has been some evidence indicating that these drugs may be responsible for sudden sensorineural hearing loss.

We also report that it is of fundamental importance to assess the mechanism of action of these drugs, to explain the possible causal association between their use and sensorineural hearing loss development, that even after the use of combination therapy (oral steroids and intratympanic injections), has been irreversible.

Based on these results, we notice that in the two cases described, the sudden sensorineural hearing loss was unilateral, accompanied by tinnitus and dizziness. Patient 1 had hearing loss in all frequencies tested, and patient 2 had greater hearing loss in the higher frequencies. These findings are consistent with the literature, as described in a histopathological study of the temporal bone, after an episode of sudden idiopathic hearing loss, which revealed a large loss of cochlear neurons, mainly in the basal turn of the cochlea, explaining the hearing loss in the high frequencies^11^.

Tinnitus represents a common complaint, perhaps the one most often associated with sudden hearing loss7., 8., 9., and we demonstrated that the patients described in this study had this symptom. It is important to notice that, despite the lack of improvement in the hearing thresholds of Patient 1 after combination treatment, there was some improvement vis-à -vis the intensity and discomfort caused by tinnitus (Tables 1 and 2), which reinforces the hypothesis that the tinnitus can recede or persist for longer than the hearing loss^10^.

Patient 2 showed a significant change in hearing thresholds, given that his severe hearing loss changed to mild after combined treatment, and so had the tinnitus, it was severe and went on to be mild (Tables 1 and 2). These results observed in Patient 2 were probably due to hearing improvement, emotional stabilization and habituation to tinnitus.

Given the fact that the clinical history of patients did not mention comorbidities and, still, that sudden hearing loss can be idiopathic, it was possible to hypothesize that the use of phosphodiesterase type 5 inhibitors may have contributed to such occurrence, and recent studies have shown such correlation2., 4., 5., 14..

It is of concern that young men, 37 and 43 years without other disease, using phosphodiesterase type 5 inhibitors have had this type of hearing loss, reinforcing the possible ototoxicity of this class of drugs and, in some cases, the irreversible hearing loss.

Regarding the studies published on this subject, the first case report of the use of sildenafil and sudden hearing loss in the literature was published in 2007 by researchers from India. According to the authors, sildenafil is effective for oral treatment of erectile dysfunction syndrome and has been broadly used in view of relatively easy availability of such medications, including the fact that it can be acquired from the Internet. However, the authors emphasized that many patients are not aware of the harmful effects of sildenafil and use the drug without medical supervision. The case described is of a 44-year-old male patient, who had bilateral profound sensorineural hearing loss, which occurred after ingestion of 50 mg/day of sildenafil for 15 days. The patient reported that after 15 days of drug administration, he noticed hearing loss in the right ear and after two days of this initial symptom, the same occurred to the left ear. The patient was treated with 1mg/Kg/day of prednisolone for a month and his hearing data was documented by means of pure tone audiometry, Distortion Product Evoked Otoacoustic Emission and Auditory Brainstem Response (ABR)^2^.

Still in 2007, after this case report, the FDA announced 113 hearing loss reports from patients who used PDE5i, and 84 of these cases were excluded from the number of cases for several reasons. The remaining 29 reports had a strong association between the use of PDE5 inhibitors (sildenafil, vardenafil, tadalafil) and sensorineural hearing loss. Besides hearing loss, there were still reports of tinnitus and dizziness. Given this, the FDA required manufacturers of these drugs to alert consumers about this possibility: sudden sensorineural hearing loss^5^.

The first experimental study was conducted in South Korea in 2008, in which the authors examined the hearing of lab animals that received high doses of sildenafil for 105 days and the rats were evaluated at baseline (0), on the fifth day (5), fifteenth (15), in the twenty-fifth day (25), in the thirty-fifth day (35) and the hundredth-and-fifth day (105). To assess the hearing, the authors used the Auditory Brainstem Response (ABR); middle latency auditory responses, as well as the evoked otoacoustic emissions. The authors reported that, in high doses, sildenafil caused changes to auditory evoked potentials, because it increased the latency duration of short latency potentials. The same kind of result occurred with the middle latency potentials. Otoacoustic emissions differ between the groups of high-dose sildenafil and the control group with long-term treatment. The authors stated that these results indicated a decrease in the number of outer hair cells of the organ of Corti and that the probable cause of this is the toxic effect due to excess nitric oxide in the auditory organs such as the cochlea and the auditory nerve. Based on these results, the authors demonstrated that the administration of large doses of sildenafil in the long term, may contribute to the development of sensorineural hearing loss^6^.

In 2009, two more cases were reported and there was also a review of post-marketing data from the FDA, through a retrospective analysis and found the following results: of 25 patients analyzed, 15 (88%) had hearing loss within 24 hours after taking PDE5 inhibitor. Eight patients (32%) reported dizziness associated with hearing loss. In 96% of reported cases, the hearing loss was unilateral. Complete hearing recovery was observed in five patients (20%), while the other three patients (12%) had at least a partial improvement. Therefore, of the 15 patients with initial hearing loss, only eight patients (32%) reported improvement compared to the initial complaint. The authors concluded that, although the data was not conclusive, there seems to be a potential link between the use of PDE5 inhibitor and sudden sensorineural hearing loss. Thus, the authors stressed that otolaryngologists should include it in their anamneses and among possible causes of sudden sensorineural hearing loss, an inquire about the use of any of the PDE-5 inhibitors. Although there is currently no direct evidence of a mechanism behind this side effect, the authors postulated that it is related to the prolonged effects of cyclic guanosine monophosphate (GMPc) within the cochlea^4^.

In 2009, the first prospective observational study was carried out in humans. Eighteen patients who were using a PDE5 inhibitor for erectile dysfunction treatment were studied. Threshold Audiometry was performed in all patients at frequencies from 250 to 16,000 Hz before and after 1, 5 and 72 hours after ingestion of 10 mg of vardenafil. Four patients showed a statistically significant increase in the hearing threshold in one ear, consistent with the criteria of ototoxicity within 72 hours of ingestion of the drug. Furthermore, all patients showed a significant unilateral decrease in the threshold at 10,000 Hz. However, with the discontinuation of the drug, there was improvement in hearing thresholds and all the hearing loss disappeared^12^.

In an epidemiological study carried out in 2010, the researcher compared subjects with and without hearing loss, who were using the PDE5i. The author points out that this was the first epidemiological study that evaluated the association between the use of PDE5 inhibitors and sensorineural hearing loss. The cohort consisted of 11,525 men aged 40 years or older and chosen from the Medical Expenditure Panel Survey between 2003 and 2006. During a period of two years, participants provided data in five interviews about their demographic information, health, healthy postures and drug prescription. They also reported no difficulty hearing or the use of hearing aids. The results showed that the incidence of hearing loss was 17.9% - a percentage which increased with age. Approximately 2% of the patients had taken some kind PDE5i, and 80.3% of them used sildenafil (Viagra).

The results showed that the hearing loss was twice as common among those who made use of PDE5 inhibitors (3.0% versus 1.4%). No significant hearing loss was seen with tadalafil or vardenafil. However, the author attributed this result to the fact that the limited use of tadalafil and vardenafil by the participants may have limited their ability to find a possible association with hearing impairment. The author also highlighted that the relationship between the use of PDE5i and hearing loss can result from a tendency of drugs that promote the congestion of nasal erectile tissue, raising middle ear pressure, as well as concerns that PDE5i works by blocking cGMP breakdown and its buildup induces gene expression through transcription factor protein phosphorylation by specific kinases, which have been associated with damage to the hair cells of the cochlea. Finally, the author points out that the irreversible nature of hearing loss and its impact on quality of life underscores the need for more research on the etiological role of PDE5i use, and because it was a cross-sectional study, it provides only a limited view on the relationship between the use of PDE5i and hearing loss; and therefore requiring further investigation^13^.

In 2010 there was a report on a patient who developed unilateral sensorineural hearing loss of sudden onset, possibly related to the use of vardenafil for erectile dysfunction treatment. They described their case study as a patient of 57 years who came to the ER with hearing loss from mild to moderate in the right ear, in the range 500–3000 Hz, confirmed by pure tone audiometry and occurred after ingestion of vardenafil. The authors described that such patient was hospitalized two days later for the administration of intravenous dexamethasone, followed by oral prednisone and an improvement in hearing occurred on the fourth day of hospitalization and he was discharged three days later, continuing prednisone in an outpatient basis. Audiometry performed 10 days after treatment with corticosteroids, confirmed that the hearing in the range of 500–3000 Hz was within normal limits.

The authors considered that there was a possible association of sudden sensorineural hearing loss associated with the consumption of vardenafil. The authors also undertook an analysis of cases of hearing loss associated with PDE5i in the United States with the FDA's Adverse Events Notice System database, to compare the characteristics of their patient with those of other cases of adverse events reported and based on the temporal relationship of sudden sensorineural hearing loss and the use of vardenafil by this patient, they propose that vardenafil is a probable cause of hearing loss. According to the authors, this case provided new evidence that the use of PDE5 inhibitor should be considered as a possible cause in patients with sensorineural hearing loss of sudden onset^3^.

A study was conducted in 2011 in order to review and analyze the current literature on the correlation between the use of PDE5i and hearing loss and suggests possible physiological mechanisms, as well as investigate the global communication of this side effect. The authors pointed out that the use of sildenafil and other PDE5i has grown rapidly since its launch in 1998 and a growing body of evidence indicates significant morbidity associated with the side effects brought about by this class of drugs vis-à -vis hearing loss.

For this study, pharmaceutics surveillance agencies in North America, Europe and Australia were contacted and were requested to provide reports of hearing loss associated with the use of PDE5i. Thereafter, the reports were examined to exclude those for whom there were other possible causes to justify the hearing loss. The authors found 47 cases of sensorineural hearing loss with a temporal association with the intake of PDE5 inhibitors, based on the literature and on data obtained from pharmaceutic surveillance agencies. Patients had a mean age of 56.6 years. Eighty-eight percent of unilateral hearing loss were reported with a uniform distribution between the left/right sides. Hearing loss occurred within 24 hours after ingestion of a PDE5 inhibitor in 66.7% (n = 18) of the cases, and the use of sildenafil accounted for over 50% of the cases. Given these results, the authors concluded that there is growing evidence that PDE5 inhibitors may induce sensorineural hearing loss. And on these results, there needs to be more awareness of this disabling side effect among the health professionals responsible for prescribing this medication. The authors also emphasize that with the expiration of sildenafil patents between 2011–2013, generic drugs are more affordable and may cause a significant increase in the use of these drugs, as well as an increase in the number of sensorineural hearing loss cases^14^.

In Turkey, in 2012, researchers analyzed the histopathological effects of sildenafil use in the long term on the cochlea in an animal model. For that, the authors studied adult male Wistar albino rats. The control group was fed with a standard laboratory diet. The study group received sildenafil 1.5 mg/kg once daily orally for 45 days. Each temporal bone was dissected and the cochleae were removed en bloc. The rats were divided into two experimental groups: Group 1 (n = 10) was the control group, to which no medication was given, and Group 2 (n = 10) received sildenafil. They used 50 mg of sildenafil (Viagra tablets, Pfizer), ground into fine powder and dissolved in 0.9% NaCl. The dose of sildenafil citrate administered in this study was 1.5 mg/kg, a dose compatible with the doses normally used in humans. Sildenafil therapy was administered orally via an orogastric tube, once a day for 45 days, until 12 hours prior to surgery. To evaluate their hearing, the authors used the Transient Evoked Otoacoustic Emissions (TEOAE) test. The authors observed that the hematoxylin and eosin stain showed no difference between the groups. With respect to immunohistochemical analysis, caspase-3 immunostaining was observed in the group taking sildenafil. In the control group, the caspase 3 immunoreactivity was not observed. Thus, the authors concluded that caspase-3 immunostaining in the sildenafil group was strongly associated with an increase in apoptotic events in the cochlea, and the long-term use of sildenafil may cause hearing loss by increasing apoptosis^15^.

In 2013, we performed the first prospective observational study using tadalafil in the auditory function, through an objective hearing evaluation. It was a prospective observational study in a tertiary hospital. Twenty-five patients who had not used tadalafil and had erectile dysfunction and pulmonary arterial hypertension underwent pure tone audiometry and Evoked Auditory Brainstem Response (ABR) prior to drug therapy, and 3 and 30 days after drug therapy. The authors found that 15 patients used 10 mg of tadalafil for erectile dysfunction, and 10 patients were taking 20 mg of tadalafil once daily for pulmonary arterial hypertension. There was no statistically significant difference in the hearing threshold at baseline and at follow-up (*p* > 0.05). However, three patients used 20mg of tadalafil and had significant increase in their hearing thresholds in the higher frequencies. There was no incidence of sudden sensorineural hearing loss in the study group.

The authors stated that this was the first prospective observational study that evaluated the effect of tadalafil on the auditory function with objective evidence. Although they did not find a statistically significant result to confirm or refute the association between tadalafil and hearing impairment, raising the threshold at higher frequencies after the use of tadalafil supports the results of previous studies and suggests a possible relationship between the two. Finally, the authors suggest that further studies should be conducted with larger similar samples to understand the exact impact of type 5 phosphodiesterase inhibitors on auditory functions^16^.

## CONCLUSION

The increased occurrence of sudden hearing loss in patients using PDE5i and scientific reports in the literature suggest that the use of phosphodiesterase type 5 inhibitors is regarded as a risk factor for hearing, indicating the need for further studies on the possible ototoxicity of this class of drugs.

## References

[1] Leong AC, Fairley JW, Padgham ND. (2007). Sudden hearing loss. Clin Otolaryngol.

[2] Mukherjee B, Shivakumar T. (2007). A case of sensorineural deafness following ingestion of sildenafil. J Laryngol Otol.

[3] Snodgrass AJ, Campbell HM, Mace DL, Faria VL, Swanson KM, Holodniy M. (2010). Sudden sensorineural hearing loss associated with vardenafil. Pharmacotherapy.

[4] Maddox PT, Saunders J, Chandrasekhar SS. (2009). Sudden hearing loss from PDE-5 inhibitors: A possible cellular stress etiology. Laryngoscope.

[5] Phosphodiesterase type 5 (PDE5) inhibitors [sildenafil citrate (marketed as Viagra and Revatio), vardenafil hydrochloride (marketed as Levitra), and tadalafil (marketed as Cialis)]: Sudden Hearing Loss. [online]. Food and Drug Administration (FDA) [Acessado em 06 de junho de 2013]. Disponível em: http://www.fda.gov/Drugs/DrugSafety/DrugSafetyNewsletter/ucm119034.htm#pde5

[6] Hong BN, Yi TH, Kim SY, Kang TH. (2008). High dosage sildenafil induces hearing impairment in mice. Biol Pharm Bull.

[7] Caldas N, Caldas SN., Lopes OF, Campos CA. (1994). Tratado de Otorrinolaringologia.

[8] Maia RA, Cahali S. (2004). Surdez súbita. Rev Bras Otorrinolaringol.

[9] Penido NO, Ramos HVL, Barros FA, Cruz OLM, Toledo RN. (2005). Clinical and etiological factors and evolution of hearing in sudden deafness. Braz J Otorhinolaryngol.

[10] Shuknecht HF. (1993). Pathology of the Ear.

[11] Bahmad F. (2008). Temporal bone histopathology - idiopathic sudden hearing loss. Braz J Otorhinolaryngol.

[12] Okuyucu S, Guven OE, Akoglu E, Uçar E, Dagli S. (2009). Effect of phosphodiesterase-5 inhibitor on hearing. J Laryngol Otol.

[13] McGwin G (2010). Phosphodiesterase type 5 inhibitor use and hearing impairment. Arch Otolaryngol Head Neck Surg.

[14] Khan AS, Sheikh Z, Khan S, Dwivedi R, Benjamin E. (2011). Viagra deafness—sensorineural hearing loss and phosphodiesterase-5 inhibitors. Laryngoscope.

[15] Bakir S, Firat U, Gün R, Bozkurt Y, Yorgancilar E, Kiniş V (2012). Histopathologic results of long-term sildenafil administration on rat inner ear. Am J Otolaryngol.

[16] Thakur JS, Thakur S, Sharma DR, Mohindroo NK, Thakur A, Negi PC. (2013). Hearing loss with phosphodiesterase-5 inhibitors: a prospective and objective analysis with tadalafil. Laryngoscope.

